# Factors Influencing Dechlorane plus Distributions in Various Sheep Tissues

**DOI:** 10.3390/ijerph19138004

**Published:** 2022-06-29

**Authors:** Hongli Jin, Wenming Chen, Junsong Bao, Te Bu, Tianwei Li, Yiming Liu, Yanli Liu, Jun Jin

**Affiliations:** 1 College of Life and Environmental Sciences, Minzu University of China, Beijing 100081, China; honglijin1998@126.com (H.J.); chenwm99@126.com (W.C.); 13398699946@163.com (J.B.); bute.bu20@imperial.ac.uk (T.B.); 18811387553@163.com (T.L.); liuym2029@stu.pku.edu.cn (Y.L.); 2 Department of Biomedicine, Beijing City University, Beijing 100094, China; 3 Beijing Food and Environmental Health Engineering Center, Beijing 100081, China

**Keywords:** Dechlorane plus, tissues and organs, fluorescence spectrometry, sheep serum albumin

## Abstract

Dechlorane plus (DP) is a potential persistent organic pollutant and its distribution in various tissues and organs of terrestrial organisms is currently unknown. DP concentrations in sheep tissues were determined in this study. The DP concentrations in the tissues decreased in the following order: abdominal fat > liver > stomach > heart > outer tenderloin > lung > hind leg meat > kidney > small intestine > tail fat > spleen > brain. Apart from brain and fat, anti-DP is enriched more readily than syn-DP in sheep tissues, but syn-DP is more readily enriched in brain and abdominal fat. The factors influencing DP distributions in sheep tissues were assessed by determining the DP to sheep serum albumin binding forces, binding types, and binding sites by fluorescence spectroscopy. The results indicated that anti-DP more readily binds to sheep serum albumin than does syn-DP. Therefore, sheep serum albumin will more readily transport anti-DP than syn-DP to sheep tissues, and anti-DP will be enriched more than syn-DP in the tissues. The molecular diameter of DP is the main factor affecting DP concentrations in sheep brain and fat because of the blood–brain barrier and because the main source of DP to abdominal fat is dermal contact.

## 1. Introduction

Dechlorane plus (DP), a substitute for mirex, was produced by a company called Hooker in the 1960s. DP is a highly chlorinated flame retardant that is cheap and has a low density, as well as good thermal and photochemical stabilities [1,2]. DP is added to many materials made from polymers, such as cable coatings, computer cases, electronic components, nylon products, and televisions [3]. DP has similar structural characteristics to many persistent organic pollutants and is very lipophilic and not readily photolyzed or biodegraded. DP can accumulate in biota [1]. DP has been classed as a suspected persistent organic pollutant under the Stockholm Convention on Persistent Organic Pollutants. DP can persist in environmental media and organisms and has expected half-lives in water, sediment, and fish of 24, 17, and 14a, respectively. Long-term exposure to DP can cause great harm to biota. Subacute toxicological experiments using DP have indicated that long-term dermal contact with or inhalation of DP at high concentrations can cause lesions in the lungs, liver, and reproductive system [4].

It has previously been found that DP has the typical characteristics of persistent organic pollutants [5,6,7]. DP is ubiquitous in the environment because it has been widely used as a flame retardant around the world for decades [8,9,10]. Few studies of DP in biological samples have been performed. Kang et al. [11] determined DP concentrations in fish samples from 15 urban areas and seven rural areas in South Korea and found that the DP concentration were 25 times higher in fish from urban areas than in fish from rural areas. In particular, DP was found at much higher concentrations in fish from industrial areas than in fish from other areas. This indicated that human activities in urban and industrial areas increase DP concentrations in the environment. Tomy et al. [2] determined DP concentrations in biota from Lake Ontario and Lake Winnipeg and detected DP in most of the organisms that were analyzed. They found that DP bioaccumulates and that the anti-DP concentrations in biota increased slightly as the trophic level increased. Wu et al. [10] determined DP concentrations in biota from various trophic levels from a reservoir near an e-waste dismantling site in South China and found that anti-DP was metabolized faster. However, syn-DP was absorbed more effectively as the trophic level increased. The syn-DP concentrations in biota increased slightly as the trophic level increased. This contradicted the results found by Tomy et al. [2] for biota in Lake Ontario and Lake Winnipeg. Tomy et al. [7] exposed salmon to DP at below the lethal concentration in water and investigated absorption and metabolism of anti- and syn-DP by the salmon. They found a higher absorption rate and a slower metabolism rate for syn-DP than anti-DP. However, few such studies have been performed. More data are needed to allow the fate of DP in the food chain to be assessed and differences in the degrees to which anti- and syn-DP bioaccumulate to be identified.

Different anti- and syn-DP concentrations may be found in different tissues of animals, and these differences can be identified using the variable *f_anti_,* defined as the proportion of total DP contributed by anti-DP. Differences between anti- and syn-DP distributions in different tissues of organisms have been found in previous studies. Zhang et al. [12] analyzed two different types of bottom-dwelling fish (dace and *Ophiocephalus argus*) from a pond at an e-waste dismantling site in South China and found that the relative anti-DP concentrations in the tissues of both fish decreased in the order brain > liver > muscle. Yin et al. [13] investigated anti- and syn-DP accumulation in rat liver, muscle, and serum and found that DP accumulated preferentially in the liver rather than muscle at all of the DP concentrations the rats were exposed to. No marked stereoselectivity of anti- or syn-DP was found in the tissues of rats exposed to DP at a low concentration. However, the *f_anti_* values were markedly lower for rats exposed to DP at a high concentration than at a low concentration, and syn-DP was the dominant DP isomer in all of the tissues of the rats exposed to DP at a high concentration. Due to the more complex environment to which terrestrial organisms are exposed, there are relatively few studies on the selective distribution of DP isomers in different tissues and organs of mammals in terrestrial ecosystems.

Serum albumin (SA) is the most abundant carrier protein in blood. When a pollutant enters the blood, it can bind to SA and be transported to tissues throughout the organism. The ability of a pollutant to bind to SA will directly affect absorption, distribution, metabolism, and excretion of the pollutant [14]. The different concentrations of anti- and syn-DP in different animal tissues may be related to the abilities of anti- and syn-DP to bind to SA and/or anti- and syn-DP binding to different sites on SA. In the study described here, anti- and syn-DP concentrations in different sheep tissues were determined and the abilities of anti- and syn-DP to bind to sheep SA (SSA) were assessed. The aim was to try to explain the differences in the distribution and enrichment of anti-DP and syn-DP in different tissues, which may help explain the transport and metabolism of DP in different organisms.

## 2. Materials and Methods

### 2.1. Chemicals

Standards of anti-DP (catalog number: a-DP), ^13^C-labeled syn-DP (catalog number: Ms-DP), and syn-DP (catalog number: s-DP) (each at 50 mg·mL^−1^, >95% pure) were purchased from Wellington Laboratories (Guelph, ON, Canada). All organic solvents were obtained from J.T. Baker (Phillipsburg, NJ, USA) and were pesticide analysis grade. Ultra-pure water was produced using a Milli-Q system (EMD Millipore, Billerica, MA, USA). Analytical grade anhydrous sodium sulfate was baked at 450 °C for 5 h before use. Silica gel (100–200 mesh; Merck, Darmstadt, Germany) was activated at 105 °C for 12 h, cooled, and deactivated by adding 3% of the sorbent weight of deionized water.

### 2.2. Sample Preparation

Tissue samples were collected from sheep kept 100 km northeast of Xilingol City, Inner Mongolia Province, China. The sheep tissue samples that were analyzed for DP were all from the same sheep. A 2.0 g aliquot of a freeze-dried sheep tissue sample was ground to a powder in a mortar with an appropriate amount of anhydrous sodium sulfate to act as an abrasive. The sample was transferred to an extraction vessel, then 4.0 ng of ^13^C-labeled *syn*-DP was added and the sample was mixed well. A 1:1:1:1 (*v*/*v*/*v*/*v*) mixture of hydrochloric acid, isopropanol, *n*-hexane, and methyl tert-butyl ether was added to the sample, then the sample and solvent were mixed well and centrifuged at 12,000 revolutions/min for 5 min. The organic phase was transferred to a glass tube containing 4.0 mL of 1% (*w*/*w*) KCl (aq). The mixture was shaken, then the organic phase was transferred to a previously weighed container. The KCl solution was extracted twice using 3.0 mL of a 1:1 (*v*/*v*) mixture of *n*-hexane and methyl tert-butyl ether. The three organic phases were mixed and evaporated to dryness. The container containing the dried extract was weighed to determine the weight of the extract. The residue was redissolved in 4.0 mL of *n*-hexane, then 2.0 mL of 0.5 mol/L KOH was added. The organic phase was transferred to a new container and evaporated to a small volume. The sample was added to a gel permeation chromatography column, which was eluted with 250 mL of a 1:1 (*v*/*v*) mixture of *n*-hexane and dichloromethane. The sample was rotary evaporated to 1 mL and finally evaporated under a stream of nitrogen to 100 µL ready for analysis by gas chromatography mass spectrometry.

### 2.3. Instrumental Analysis

The extracts were analyzed using an Agilent 6890 gas chromatograph and Agilent 5975n mass spectrometer (Agilent Technologies, Santa Clara, CA, USA). The mass spectrometer was used in negative chemical ionization mode and selective ion monitoring mode. The carrier gas was helium; the flow rate was 1.0 mL/min. The reagent gas was methane and the flow rate was 1.0 mL/min. The syringe, mass spectrometer source, and quadrupole temperatures were 250, 150, and 150 °C, respectively. The gas chromatograph was equipped with a J&W DB5-ms column (30 m long, 0.25 mm inner diameter, 0.25 m film thickness; Agilent Technologies). The oven temperature program started at 100 °C, which was held for 3 min, then increased at 10 °C/min to 300 °C, which was held for 22 min. The *m*/*z* ratios monitored for unlabeled DP were 652.0 and 654.0, and the *m*/*z* ratios monitored for ^13^C-labeled DP were 666.0 and 668.0.

At room temperature, 10 mL of a SSA solution at a concentration of 10^−6^ mol/L was added to each of three 25 mL colorimetric tubes, then 2.0 × 10^−3^ mol/L syn-DP was added to one tube, 2.0 × 10^−3^ mol/L anti-DP was added to another tube, and nothing was added to the third tube. The fluorescence spectra of the solutions were acquired at room temperature using an f-4500 fluorescence spectrophotometer (Hitachi High-Technologies, Tokyo, Japan), and the fluorescence intensities were determined. The slit width was 5 nm, the voltage was 700 V, and the scanning speed was 1200 nm/min. The excitation wavelength was 280 nm. Fluorescence spectra in the range 300–500 nm were acquired.

### 2.4. Quality Assurance and Quality Control

The target compounds were quantified using an internal standard method using the isotope-labeled internal standard. The limit of quantification was defined as the concentration giving a signal-to-noise ratio of 10. The correlation coefficients of the standard calibration curves for the analytes were >0.9996. The mean ^13^C-labeled syn-DP recovery was 84.1 ± 27.6%. The lower quantitation limits for anti- and syn-DP were 0.17 and 0.2 ng/g, respectively. A method blank was analyzed with each batch of samples. The analyte concentrations in the blank samples were very low (<10% of the concentrations in the samples), and the signal-to-noise ratios were <10, so the concentrations in the samples were not blank corrected. Each fluorescence measurement was repeated three times using different conditions, then the mean fluorescence intensity was calculated. The DP concentrations in the organs or tissues of sheep were detected by three flat tests and the average value was taken.

### 2.5. Software

Linear fitting and histogram analyses were performed using Microsoft Excel 2010 software. Molecular docking between the clone and CYP3A24 was simulated using AutoDockTools molecular docking software (Version 1.5.6, Clinton Township, MI, USA, Olson Lab) (https://ccsb.scripps.edu/mgltools/downloads/ (accessed on 12 March 2022)).

## 3. Results and Discussion

### 3.1. Concentrations of DP in Sheep Tissues

**Figure 1:**
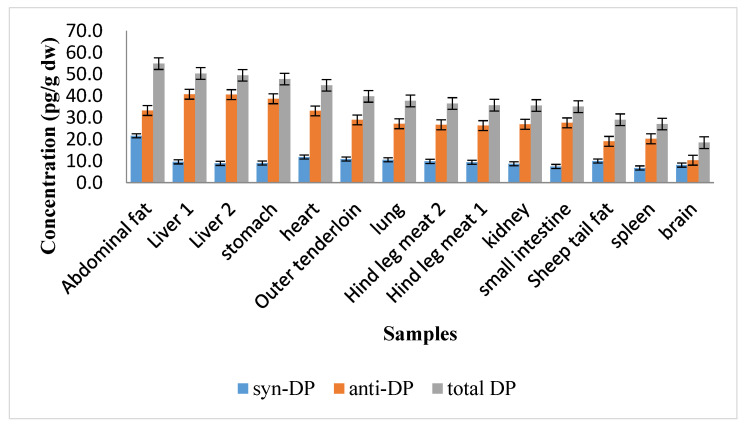
Concentrations of syn-Dechlorane plus (DP), anti-DP, and total DP in various sheep tissues.

The DP concentrations found in the sheep tissue samples are shown in Figure 1. The total DP concentrations in the sheep tissue samples ranged from 18.4 to 54.9 pg/g dry weight (dw), and the mean concentration was 38.7 pg/g dw. The highest DP concentration in abdominal fat, 54.9 pg/g dw, was higher than the DP concentrations in the other tissues. The DP concentration was much higher in abdominal fat than tail fat (29.0 pg/g dw). This indicated that the DP concentration was lower in tissue with a lower degree of blood perfusion, i.e., tail fat. The DP concentration was lower in the sheep brain (18.4 pg/g dw) than the other tissues. The DP concentrations in the sheep tissue samples decreased in the following order: abdominal fat > liver 2~liver 1 > stomach > heart > outer tenderloin > lung > hind leg meat 1~hind leg meat 2 > kidney > small intestine > sheep tail fat > spleen > brain.

Zheng et al. [15] found that more DP accumulated in fat than other organs of chickens and concluded that DP is more enriched in fat than in other tissues. Among all chicken tissues analyzed, fat accounting for 56% of the total amount DP in all of the chicken tissues that were analyzed. The tissues that accumulated the next largest amounts of DP were liver (12% of the total amount of DP) and gonads (12% of the total amount of DP). These results were consistent with our finding that the DP concentration was higher in abdominal fat than other sheep tissues. This agreed with the DP concentration being higher in abdominal fat than other sheep tissues in our study. The skin on the abdomen of a sheep is often in contact with environmental media (e.g., soil), so a large proportion of DP in abdominal fat may be supplied through dermal contact with soil and grass. Rang et al. [16] suggested that ingested pollutants are first deposited in the central tissues that are abundantly perfused with blood and then can be transferred to the peripheral tissues. Fat is poorly perfused with blood, meaning less blood reaches fat than other tissues. Li et al. [17] exposed rats to food containing DP and found higher DP concentrations in liver than muscle. These results were also consistent with the results of our study. The liver is the main organ in sheep in which metabolism occurs, but the ease with which DP is metabolized in vivo is not yet clear. Digestion in sheep mostly occurs in the stomach. Inhaled air enters the lungs. High DP concentrations in the stomach may be caused by direct contact with food and soil particles containing DP. DP may enter the lungs through inhalation of DP in the particulate or vapor phase. The DP concentration was lower in the brain than the other tissues, probably because of the blood–brain barrier.

### 3.2. Binding between DP and SSA

Similar to most persistent halogenated compounds, the distributions of DP in tissues is controlled by distribution between lipid pools and is also related to the transport of DP bound to SA and DP binding to specific proteins in the liver. Analyzing the ability of DP to bind to SSA is, therefore, needed to improve our understanding of DP transformation mechanisms.

#### 3.2.1. Fluorescence Quenching

**Figure 2:**
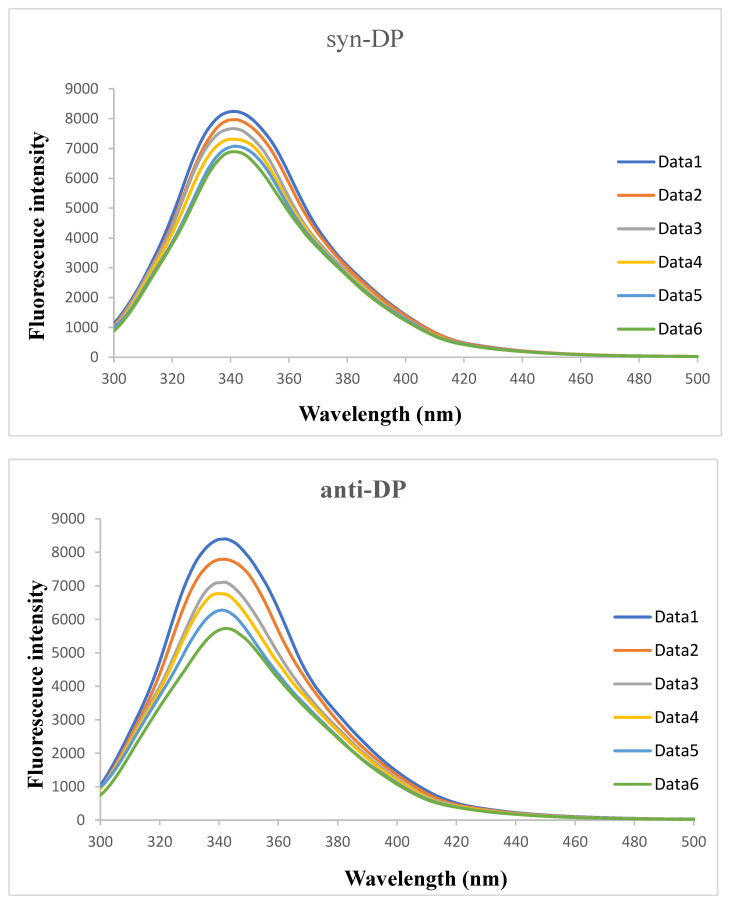
Fluorescence quenching curves for sheep serum albumin in the presence of syn-Dechlorane plus (DP) and anti-DP at room temperature (Data1, Data2, Data3, Data4, Data5, and Data6 are for DP concentrations of 0, 2 × 10^−7^, 4 × 10^−7^, 6 × 10^−7^, 8 × 10^−7^, and 10 × 10^−7^ mol/L, respectively).

Fluorescence quenching means that the fluorescence emission intensity of a fluorescent substance is decreased or extinguished by a quenching substance. The fluorescence intensities of mixtures of SSA and anti- and syn-DP at different concentrations were measured using a fluorescence spectrophotometer. The fluorescence quenching curves for anti- and syn-DP interacting with SSA at room temperature are shown in Figure 2. 

Yang et al. [18] studied interactions between Matrine and human SA and bovine SA and found that endogenous fluorescence of SA was mainly caused by phenylalanine, tryptophan, and tyrosine residues. The fluorescence intensity of phenylalanine residues near 284 nm was found to be weakest, the fluorescence intensity of tyrosine residues near 304 nm was next weakest, and the fluorescence intensity of tryptophan residues near 335 nm was found to be strongest. Matrine interacts with human SA and bovine SA to form a complex in the ground state. Formation of the complex quenches human SA and bovine SA fluorescence. Figure 2 shows that there was a strong fluorescence emission peak at 341 nm when SSA was in the presence of anti- or syn-DP. This indicated that fluorescence quenching may occur when anti- or syn-DP combines with tryptophan in SSA. The fluorescence intensity of the SSA gradually decreased as the DP concentration increased, indicating that anti- or syn-DP can interact with SSA to form a new species that does not fluorescence or weakly fluoresces.

#### 3.2.2. Binding Constant for DP and SSA

There are three main types of fluorescence quenching, dynamic quenching, static quenching, and energy transfer [19]. The mechanism involved in SSA fluorescence quenching by DP was investigated. Equation (1) was used to calculate the bimolecular quenching rate constant K_q_.

F_0_/F = 1 + K_q_ι_0_[Q] = 1 + [Q]K_sv_ (1)

**Figure 3:**
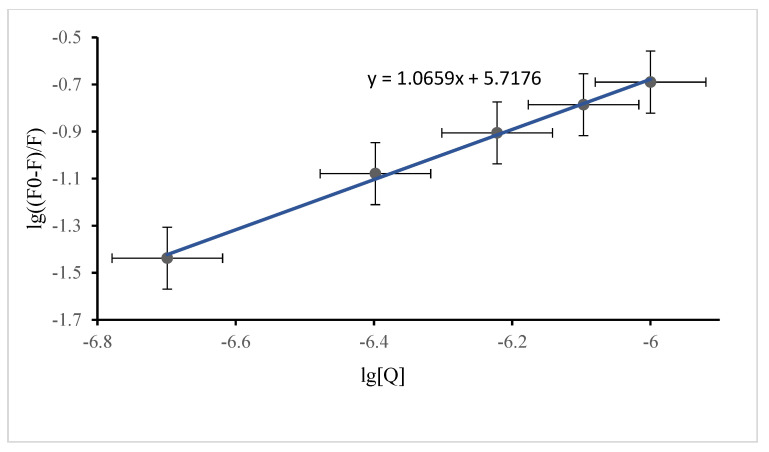
Double logarithm quenching plot for syn−Dechlorane plus and sheep serum albumin.

**Figure 4:**
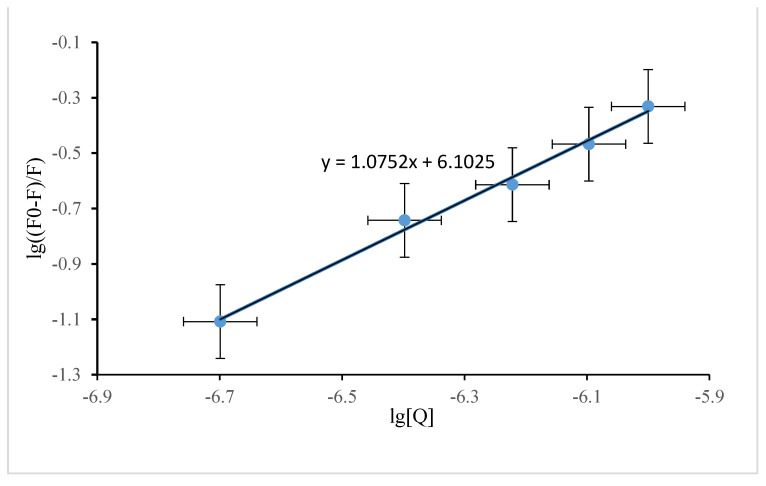
Double logarithm quenching plot for anti−Dechlorane plus and sheep serum albumin.

In Equation (1), F_0_ is the SSA fluorescence intensity (8241 and 8390) at 341 nm without anti- or syn-DP present, F is the fluorescence intensity of the solution with anti- or syn-DP present at a concentration of 2 × 10^−7^ mol/L (fluorescence intensities 7965 and 7789, respectively), K_SV_ is the quenching constant, K_Q_ is the dynamic bimolecular quenching rate constant, and ι_0_ is the fluorescence lifetime of the fluorescent substance [13,20,21]. The endogenous fluorescence lifetime of a general biological protein, 10^−8^ s, was used [22]. Stern–Volmer diagrams of SSA fluorescence quenching by anti- and syn-DP were plotted with [Q] on the *x*-axis and F_0_/F on the *y*-axis. K_SV_ for syn-DP was found to be 1.83 × 10^4^ and K_SV_ for anti-DP was found to be 4.43 × 10^4^. K_q_ for syn-DP was found to be 1.83 × 10^12^ L/(mol s) and K_q_ for anti-DP was found to be 4.43 × 10^12^ L/(mol s). The bimolecular quenching rate constants K_q_ were much higher than 2.0 × 10^10^ L/(mol s), indicating that anti- and syn-DP caused static quenching of SSA fluorescence [23]. The data were processed using Equation (2) for static quenching. During interactions between a small molecule and a biological macromolecule, the binding constant K_a_, the number of binding sites n, and the concentration of the fluorescence quencher [Q] conform to the Lineweaver–Burk equation [24].

(2) $$\mathrm{lg}\;(\frac{F_{0}-F}{F})={\mathrm{lgK}}_{a}+\mathrm{nlg}\left[Q\right]$$

where F_0_ is the fluorescence intensity of SSA without DP present, F is the fluorescence intensity of SSA with DP present, [Q] is the DP concentration, lgK_a_ is the intercept, and n is the slope. Plots of lg (F_0_ − F/F) against lg [Q] for syn-DP and anti-DP are shown in Figure 3 and Figure 4, respectively. The binding constant for syn-DP binding to SSA was found to be K_a (*syn-*DP)_ = 10^5.72^, n = 1, and the binding constant for anti-DP binding to SSA was found to be K_a (*anti-*DP)_ = 10^6.10^, n = 1. It can be seen that K_a (*anti*-DP)_ was larger than K_a (*syn*-DP)_, indicating that anti-DP binds more readily to SSA than does syn-DP and, therefore, that anti-DP can more readily be stored and transported by protein within a sheep than syn-DP. n = 1 indicates that DP and SSA form a 1:1 complex that is transported within a sheep.

#### 3.2.3. Ability of DP to Bind to CYP3A24

AutoDockTools 1.5.6 molecular docking software was used to simulate molecular docking of the DP isomers with CYP3A24. Using semi-flexible docking mode, each DP isomer was randomly docked with CYP3A24 100 times. The Lamarckian genetic algorithm was used to optimize the energy [25] and the conformation with the highest score weight was selected as the final docking result. The binding free energy of syn-DP was found to be −ΔG_binding_ = 8.06 kcal/mol, and the binding free energy of anti-DP was found to be −ΔG_binding_ = 7.39 kcal/mol. The binding free energy was found to be higher for syn-DP and CYP3A24 than for anti-DP and CYP3A24. The metabolic rate may therefore be higher for metabolism of syn-DP by CYP3A24 than for metabolism of anti-DP by CYP3A24 in the liver. Li et al. [17] found syn-DP elimination half-lives of 179, 44, and 24 d for liver, muscle, and serum, respectively, in rats and anti-DP elimination half-lives of 54 and 25 d for muscle and serum, respectively. These results indicated that syn-DP is less readily eliminated from the liver than muscle or serum. The syn-DP half-life in rat liver indicates that DP is not readily metabolized in an organism. Li et al. [17] did not determine the anti-DP half-life in liver in rats. However, anti-DP is less readily metabolized than syn-DP in muscle and serum, so it would be expected that anti-DP would also be less readily metabolized than syn-DP in liver. Tomy et al. [7] found a higher decomposition rate for syn-DP (0.065 nmol/d) than anti-DP (0.024 nmol/d) in chicken liver. 

### 3.3. f_anti_ Values for Sheep Tissues

**Figure 5:**
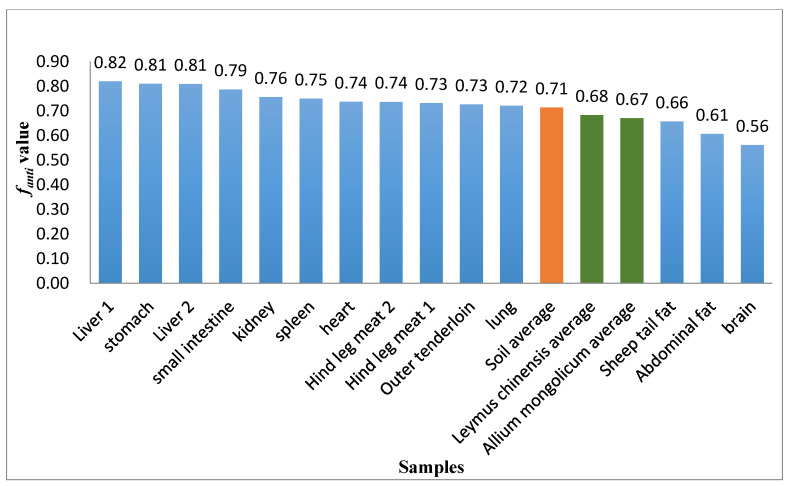
*f_anti_* values for various sheep tissues, plants, and soil.

The *f_anti_* values for the different sheep tissues ranged from 0.56 to 0.82. Figure 5 shows the *f_anti_* values. The *f_anti_* values for the tissues decreased in the following order: liver 1~liver 2 > stomach > small intestine > kidney > spleen > heart > hind leg meat 1~hind leg meat 2 > outer tenderloin > lung > tail fat > abdominal fat > brain. Chen et al. found *f_anti_* values of 0.67–0.71 for soil, *Leymus chinensis*, and *Allium mongolicum* from the area in which the sheep used in this study were kept [26]. The *f_anti_* values for liver, small intestine, and stomach (range 0.79–0.82) found in our study were higher than the *f_anti_* values for soil and grass (range 0.67–0.71), indicating that the liver, small intestine, and stomach were relatively enriched in anti-DP. The *f_anti_* values were lower for brain and abdominal fat (0.56–0.61) than for soil and grass (0.67–0.71), meaning brain and abdominal fat were enriched in syn-DP relative to anti-DP. The *f_anti_* values for heart, kidney, lung, muscle, spleen, and other tissues were similar to the *f_anti_* values for soil and grass. The *f_anti_* values for most of the tissues (except brain and abdominal fat) were similar to or markedly higher than the *f_anti_* values for environmental media in the area the sheep were kept, indicating that most sheep tissues were more likely to be enriched in anti-DP than syn-DP. It was previously found that anti-DP more readily combines with SSA than does syn-DP and that anti-DP is therefore more readily transported to various tissues by SSA and enriched in the tissues.

The *f_anti_* values were lower for brain and abdominal fat than for the environmental media. The lowest *f_anti_* was for brain (0.56), and the anti-DP concentration was lower in brain than the other tissues. Even though anti-DP more readily binds to SA than does syn-DP, anti-DP was less enriched in the brain than the other tissues. The syn-DP molecule has a diameter of 10.08 Å, which is smaller than the diameter of the anti-DP molecule (11.09 Å) but the octanol–water partition constants of anti-DP and syn-DP are the same, so the blood–brain barrier must be the main factor controlling the difference in anti-DP and syn-DP enrichment in the brain. The smaller diameter of syn-DP than anti-DP means syn-DP will pass through the blood–brain barrier more readily than will anti-DP. This explains the *f_anti_* being lower for the brain than the other tissues. The *f_anti_* value for abdominal fat was also lower than the *f_anti_* values for the environmental samples. Hoh et al. [27] suggested that syn-DP is preferentially enriched in fat in fish, which is consistent with the results of our study. Adipose tissue is not strongly perfused with blood, so DP is not readily transported to fat in sheep. DP in abdominal fat is mainly supplied through dermal exposure. The smaller molecular diameter of syn-DP than anti-DP means that syn-DP enter subcutaneous fat through the skin and are enriched more readily than anti-DP. We concluded that the anti-DP and syn-DP molecular diameters were the main factors causing the *f_anti_* values to be lower for the brain and abdominal fat than the other tissues.

The *f_anti_* value was higher for the liver (0.82) than the other tissues, indicating that anti-DP was more enriched in the liver than the other tissues. Zheng et al. [15] found *f_anti_* values for chicken tissues of 0.39–0.79. They found a higher *f_anti_* value for chicken liver than the other tissues, which was consistent with our results. The free energy of DP binding to CYP3A24 cannot determine the rate at which CYP3A24 metabolizes anti- and syn-DP in sheep liver but can at least explain why, when DP was in dissociated equilibrium in sheep liver, the equilibrium constant was higher for syn-DP and CYP3A24 than anti-DP and CYP3A24. This would cause the free anti-DP concentration in the liver to increase. This could be one of the factors causing the anti-DP concentration to be higher in the sheep liver than the other tissues.

The *f_anti_* value for industrial DP products is 0.75 [8]. Various conclusions have been drawn from the results of studies of *f_anti_* values for human serum. *f_anti_* values of 0.71–0.86 have been found for serum from the general human population (low exposure to DP) [28,29,30]. However, *f_anti_* values for serum from workers and residents of high exposure areas (such as near a DP production plant [31] and in an e-waste dismantling area [32,33] were markedly lower (0.53–0.65). This indicates that the DP concentrations humans are exposed to may strongly affect the *f_anti_* value for serum. The same pattern was found in a study of rats exposed to DP performed by Li et al. [17]. In that experiment, the *f_anti_* values for blood, liver, and muscle from rats exposed to DP at a low concentration were 0.74–0.78, but the *f_anti_* values for rats exposed to DP at a high concentration were markedly lower, 0.26–0.30 [17]. The low DP concentrations to which the sheep used in this study were exposed meant that the *f_anti_* values for the tissues other than brain and fat were higher than the *f_anti_* values for the environmental samples. We concluded that the DP concentration to which an animal is exposed will affect the *f_anti_* value for serum. The DP intake of an organism is higher in an area with a high DP concentration than in an area with a low DP concentration, so the amounts of anti- and syn-DP undergoing transport by SA or metabolism by liver enzymes are small (or even negligible) relative to the amounts of anti- and syn-DP ingested by the organism (or the amounts in the blood). The different abilities of anti- and syn-DP to bind to SA and the different abilities of enzymes to metabolize anti- and syn-DP in the liver are longer the main factors affecting the *f_anti_* values for tissues in areas with high DP concentrations. The physicochemical properties (e.g., molecular radius and octanol–water partition coefficient) of anti- and syn-DP may be the main factors affecting the *f_anti_* values of tissues in areas with high DP concentrations. 

## 4. Conclusions

In this study, the total DP concentration in various tissues and organs of sheep ranged from 18.4 to 54.9 pg/g dw. The concentration order of DP in various tissues and organs of sheep was as follows: abdominal fat > liver > stomach > heart > outer tenderloin > lung > hind leg meat > kidney > small intestine > sheep tail oil > spleen > brain. The concentration order of DP in sheep tissues and organs was related to whether the blood perfusion of tissues and organs was sufficient and whether they are directly exposed to DP. By calculating the *f_anti_* of various tissues and organs in sheep, it was found that the binding ability of syn-DP, anti-DP to sheep serum albumin was the main factor affecting *f_anti_* in the tissues and organs of sheep except fat and brain with low exposure level. In addition, the binding ability of CYP3A24 enzyme with syn-DP in liver was stronger than that with anti-DP, which led to the increase of free anti-DP in liver. Because the brain has blood-brain barrier and the DP source of abdominal fat mainly came from skin contact exposure. The molecular diameter of DP is the main factor affecting the distribution of DP in sheep brain and fat. syn-DP with smaller molecular diameter is easier to pass through the blood–brain barrier and skin, resulting in their *f_anti_* value to be lower than other tissues and organs of sheep. Taken together, the results indicated that the distribution of a pollutant in an organism can be affected by various factors, including the pollutant concentrations in the media the organism is exposed to, the physical and chemical characteristics of the pollutant, and the ability of the pollutant to bind to SA. 

## Data Availability

Not applicable.
